# Editorial: IgE mediated and non IgE mediated cow’s milk protein allergy

**DOI:** 10.3389/falgy.2023.1354711

**Published:** 2024-01-08

**Authors:** Rita Nocerino

**Affiliations:** ^1^ Department of Translational Medical Science, University of Naples Federico II, Naples, Italy; ^2^ ImmunoNutritionLab at CEINGE Advanced Biotechnologies, University of Naples Federico II, Naples, Italy

**Keywords:** nutrition, hypoallergenic formulas, food allergy, growth, symptoms

**Editorial on the Research Topic** IgE mediated and non IgE mediated cow’s milk protein allergy

Cow's Milk Protein Allergy (CMPA) poses a significant challenge in pediatric care, necessitating ongoing research to refine nutritional management strategies. This editorial embarks on a journey through the latest research contributions within this Research Topic, aiming to frame the specific objectives of each study while contextualizing their findings within the broader landscape of CMPA management.

The primary objective across the contributing articles is a collective pursuit of advancing the nutritional management of CMPA. Each study undertakes a unique angle, contributing to a more comprehensive understanding of how various nutritional interventions impact the diverse presentations of this prevalent allergy.

Estrada Reyes et al. commence the exploration by focusing on the resolution of symptoms and growth outcomes in children with CMPA in Mexico. The study's objective is to delve into the real-world impact of two hydrolyzed formulas. Beyond mere symptom relief, the researchers aim to unravel the intricate relationship between the chosen formulas and the growth trajectories of pediatric patients. By retrospectively assessing a cohort of children, the study seeks to bridge the gap between clinical efficacy and everyday applicability.

Sekkidou et al. carve out a place in the broader landscape, concentrating on infants with non-IgE-mediated CMPA enteropathies and constipation. The study's objectives extend beyond conventional symptom alleviation, aiming to understand the specific impact of a casein-based extensively hydrolyzed formula on this subset of patients. The goal is to provide tailored insights into nutritional interventions for a cohort often overshadowed in broader studies, thereby contributing to a more nuanced understanding of CMPA.

Nocerino et al. present a pivotal piece that explores body growth assessment in children with IgE-mediated CMPA fed with a novel amino acid-based formula. The research's primary aim is to investigate the potential benefits of amino acid-based formulas in promoting optimal growth. By doing so, the study seeks to expand the repertoire of nutritional strategies available for children with CMPA, emphasizing the importance of tailoring interventions to individual patient needs for sustained growth.

While each article addresses specific facets of CMPA management, their collective findings resonate within a broader context, contributing to the evolving narrative of nutritional strategies for this complex condition. The shift from a one-size-fits-all approach to personalized interventions is a recurring theme. These studies collectively challenge the notion that CMPA management can be universally standardized. Instead, they advocate for a nuanced understanding of the varied clinical presentations and response profiles of affected children.

Furthermore, the introduction of different formulas represents a paradigm shift in CMPA management. These formulas offer alternatives suggesting that innovation in nutritional science holds the key to optimizing outcomes for children with CMPA.

The studies also underscore the importance of real-world applicability. By retrospectively examining cases in Mexico, Estrada Reyes et al. bring a practical dimension to their findings, emphasizing the need for nutritional interventions that resonate with the socio-cultural context of diverse populations. This real-world perspective is crucial in ensuring that research outcomes translate into meaningful improvements in clinical practice. The dedication to refining nutritional strategies in both IgE-Mediated and Non-IgE-Mediated contexts marks a significant step forward in the quest to provide comprehensive and effective care for those grappling with the complexities of CMPA.

This editorial, therefore, serves not only as a guide through recent research contributions but as a testament to the evolving understanding and commitment towards enhancing the lives of children with CMPA. The collective pursuit of refining nutritional management strategies for CMPA is evident across the studies. As we delve into the nuanced findings of each article, it becomes clear that these studies not only enhance our understanding of CMPA but also advocate for a personalized and innovative approach to nutritional interventions.

As the landscape of pediatric allergy continues to evolve, these studies stand as pillars, supporting the paradigm shift towards tailored and effective nutritional management for children with CMPA. The ongoing dedication to unraveling the complexities of this prevalent condition reflects a commitment to improving the lives of affected children and their families.

